# The Dual Role of Low-Density Lipoprotein Receptor-Related Protein 1 in Atherosclerosis

**DOI:** 10.3389/fcvm.2021.682389

**Published:** 2021-05-28

**Authors:** Jiefang Chen, Ying Su, Shulan Pi, Bo Hu, Ling Mao

**Affiliations:** Department of Neurology, Tongji Medical College, Union Hospital, Huazhong University of Science and Technology, Wuhan, China

**Keywords:** LRP1, atherosclerosis, smooth muscle cells, macrophages, endothelial cells, adipocytes, immune cells

## Abstract

Low-density lipoprotein receptor–related protein-1 (LRP1) is a large endocytic and signaling receptor belonging to the LDL receptor (LDLR) gene family and that is widely expressed in several tissues. LRP1 comprises a large extracellular domain (ECD; 515 kDa, α chain) and a small intracellular domain (ICD; 85 kDa, β chain). The deletion of LRP1 leads to embryonic lethality in mice, revealing a crucial but yet undefined role in embryogenesis and development. LRP1 has been postulated to participate in numerous diverse physiological and pathological processes ranging from plasma lipoprotein homeostasis, atherosclerosis, tumor evolution, and fibrinolysis to neuronal regeneration and survival. Many studies using cultured cells and *in vivo* animal models have revealed the important roles of LRP1 in vascular remodeling, foam cell biology, inflammation and atherosclerosis. However, its role in atherosclerosis remains controversial. LRP1 not only participates in the removal of atherogenic lipoproteins and proatherogenic ligands in the liver but also mediates the uptake of aggregated LDL to promote the formation of macrophage- and vascular smooth muscle cell (VSMC)-derived foam cells, which causes a prothrombotic transformation of the vascular wall. The dual and opposing roles of LRP1 may also represent an interesting target for atherosclerosis therapeutics. This review highlights the influence of LRP1 during atherosclerosis development, focusing on its dual role in vascular cells and immune cells.

## Introduction

Atherosclerosis (AS), in concert with its related disorders, such as coronary heart diseases, stroke, and peripheral vascular diseases, is the leading cause of morbidity and mortality worldwide (1–4). Some researchers have characterized atherosclerosis as damage to three major processes: systemic and cellular cholesterol homeostasis, inflammation and apoptosis/endocytosis (5). Atherosclerosis is initiated by endothelial dysfunction due to activation of endothelial cells by irritative stimuli, such as hyperlipidemia, high shear forces, hypertension, and proinflammatory mediators, which allow blood monocytes to permeate the endothelial cell layer and infiltrate into the intima and subintima. After entering into the intima, the monocytes differentiate into macrophages, which subsequently uptake modified low-density lipoprotein (LDL) or oxidized phospholipids to further form foam cells. The successive accumulation of apoptotic foam cells in the endothelium cannot be cleared in time, gradually leading to the formation of thrombi and inflammatory necrotic cores (6, 7). Subsequently, the release of the chemoattractant platelet-derived growth factor from macrophages, activated platelets and endothelial cells causes vascular smooth muscle cells (VSMCs) to migrate from the medium to the intima, proliferate, undergo apoptosis and senescence, and produce extracellular matrix to form fibrous caps of atherosclerotic plaques to prevent plaques rupture. However, the aberrant proliferation of VSMCs promotes plaque formation, and the balance of VSMC proliferation between migration vs. cell death and senescence determines the population of VSMCs within the atherosclerotic plaques. These processes play important roles in the formation of atherosclerosis and the stability of plaques (8, 9). Lastly, the rupture of atherosclerotic plaques promoted by cap thinning due to the death of VSMCs and the breakdown of collagen and ECM leads to thrombosis, potentially resulting in major cardiovascular episodes such as stroke and myocardial infarction (9).

LDL receptor-related protein (LRP1) is a 600-kDa type I glycosylated transmembrane protein belonging to the LDL receptor (LDLR) superfamily and is ubiquitously expressed in multiple cell types (10–13). This multifunctional transmembrane protein has been reported to regulate cholesterol homeostasis, inflammation and apoptosis/endocytosis (14–16). LRP1 is expressed in both normal and atherosclerotic arteries (17) and can recognize both lipoprotein and non-lipoprotein ligands to participate in a wide variety of biological processes, including lipid metabolism (18), blood-brain barrier (BBB) integrity (19) and macrophage migration (20). Genome-wide association studies have revealed that the LRP1 gene constitutes a susceptibility locus for abdominal aortic aneurysms, elevated plasma lipids and coronary heart disease (21–23). Many translational studies have shown that LRP1 is involved in two major physiological processes: endocytosis and signaling pathway regulation. The endocytosis of multiple extracellular ligands of LRP1, including apolipoprotein E (ApoE)- and lipoprotein lipase-enriched lipoproteins, thrombospondin, and plasminogen activators, is important in vascular biology and tumor progression. In addition, LRP1 can initiate and regulate diverse signaling pathways, including the mitogen-activated protein kinase (MAPK), insulin receptor (IR), serine/threonine protein kinase (AKT), extracellular signal-regulated kinase (ERK), and c-jun N-terminal kinase (JNK) pathways (24–29).

However, recent studies have shown that LRP1 has dual and opposing roles in regulating arteriosclerosis. Studies have indicated that LRP1 participates in the removal of atherogenic lipoproteins and other proatherogenic ligands, such as tissue-type plasminogen activator (t-PA) and urokinase-type plasminogen activator (u-PA), from the circulation in the liver (30, 31). Hepatic LRP1 plays a protective role in atherogenesis but does so independent of plasma cholesterol. In addition, several studies have revealed that LRP1 expressed in the VSMCs (14, 32–35) and macrophages (15, 35–39) protects the vasculature against the development of atherosclerosis. Moreover, another study showed that LRP1 stimulates a canonical Wnt5a signaling pathway to prevent cholesterol accumulation in fibroblasts (40). However, several studies have revealed that LRP1 efficiently internalizes aggregated LDL (aggLDL), which binds to LRP1 cluster II, into VSMCs (41–46) and macrophages (46), causing high intracellular accumulation of cholesteryl esters (CEs) in these cells. Furthermore, LRP1 also regulates cholesterol accumulation in macrophages (47), promoting their transformation into foam cells and leading to atherosclerosis. In turn, cholesterol accumulation promotes LRP1 overexpression and induces a positive feedback loop that efficiently induces the formation of VSMC- and macrophage-derived foam cells (44, 46, 48). AggLDL, a major modified form of LDL in the arterial intima, is a potent inducer of massive intracellular cholesteryl ester accumulation in both macrophages (49–51) and VSMCs (41, 44, 48). In addition, studies have shown that LRP1 mediates cholesteryl ester accumulation from lipoproteins in cardiomyocytes (52, 53), and another study showed that LRP1 controls adipogenesis and is upregulated in obese adipose tissue from humans and mice (54). Moreover, LRP1 plays a major role in controlling brain protein and lipoprotein metabolism as well as the development and regeneration in the central nervous system (55) and can also regulate the inflammatory response in the lung (56).

In this review, we mainly provide a great detailed discussion of the dual role of LRP1 in regulating atherosclerosis and its implication in antiatherosclerosis or proatherosclerosis, in order to enhance our understanding of the underlying mechanism in order to develop novel prophylactic and therapeutic strategies against cardiovascular diseases (CVDs).

## Introduction to LRP1

### Discovery of LRP1

In 1988, Herz et al. described a cell surface protein containing 4,544 amino acids that was abundant in the liver and had high structural and biochemical similarity with the LDL receptor. They named this protein LDL receptor-related protein (LRP) (12). Later, this receptor was identified by Ashcom et al. and Moestrup and Gliemann, who isolated and sequenced the liver receptor responsible for the catabolism of the α-2-M-proteinase complex (57, 58). Beisiegel et al. described LRP as an ApoE-binding protein that plays an important role in cholesterol metabolism by mediating the uptake of LDL from plasma into cells (59). In addition, LRP is a large multifunctional clearance receptor that has been implicated in the hepatic uptake of chylomicron remnants and the removal of both circulation and extracellular space-associated protease-inhibitor complexes (60). The LRP1 gene is located on chromosome 12q13-14 (61) and is synthesized in the endoplasmic reticulum as a transmembrane glycosylated precursor protein with an apparent molecular mass of ~600 kDa. After it enters the Golgi complex, LRP1 is cleaved to generate two subunits (62). Global knockout of the LRP1 gene in mice is embryonic lethal, demonstrating an essential role for LRP1 in embryogenesis and development (60, 63).

### Structure of LRP1 and sLRP1

#### Structure of LRP1

**Figure 1:**
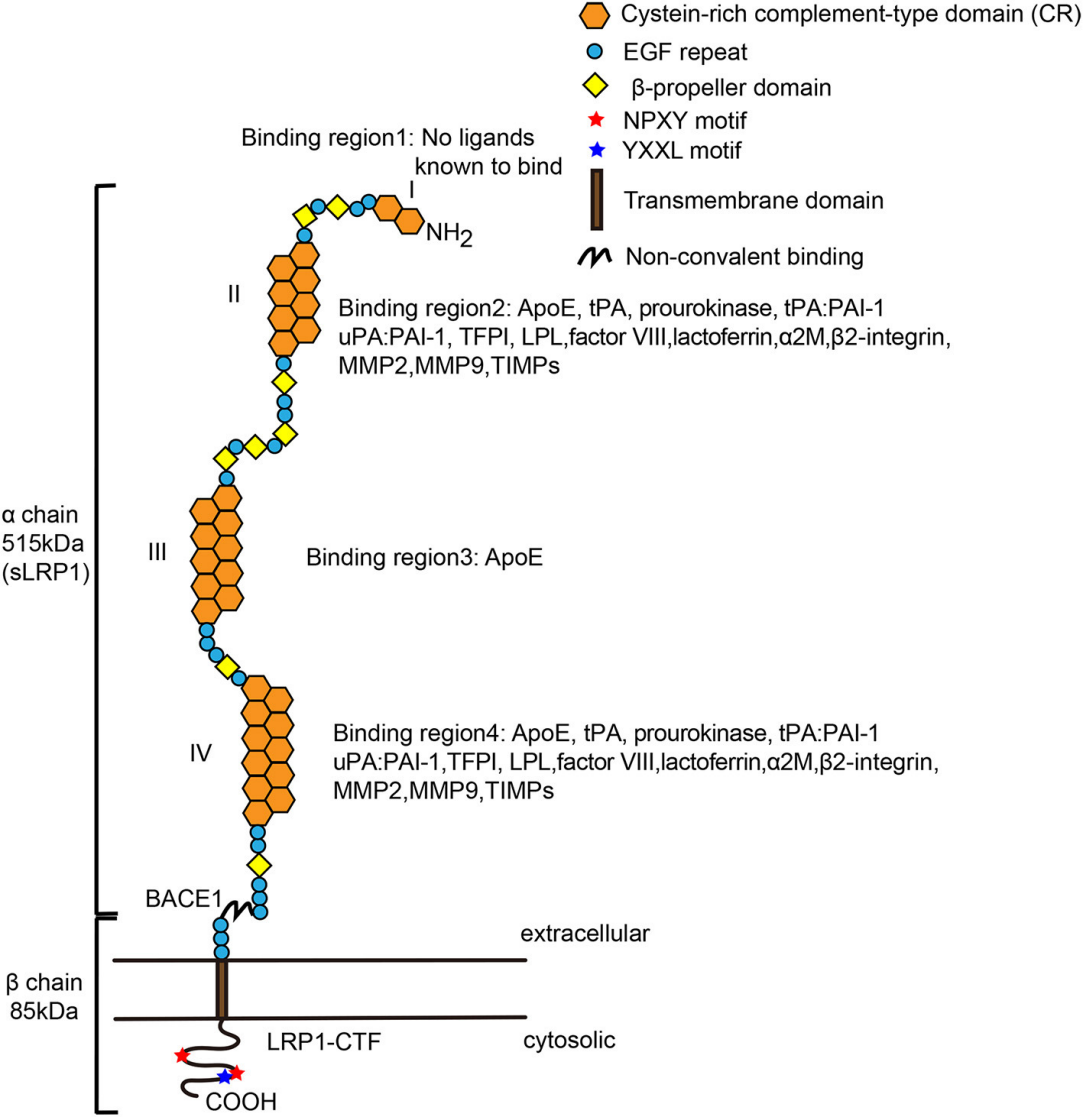
Structure of mature low-density lipoprotein receptor-related protein-1 (LRP-1). LRP1 is a multifunctional receptor that binds a large spectrum of extracellular and intracellular ligands. The α chain contains long modular arrays of acidic cysteine-rich complement-type repeats (CRs), along with epidermal growth factor (EGF)-like domains and β-propeller modules. Also, it consists of four ligand-binding regions (I, II, III, and VI), which are composed of 2, 8, 10, and 11 CRs, respectively. The cytoplasmic tail contains two NPxY motifs that are required for endocytosis and multiple signaling pathways. LRP1 has an YXXL motif adjacent to the second NPxY motif, enabling rapid endocytosis. The β chain also interacts with scaffolding proteins such as PSD-95, Dab-1, and FE-65. Regions II and IV bind most of the currently mapped known ligands of LRP1. β-Secretase (BACE1) cleaves the extracellular domain of LRP1 to form sLRP1 and LRP1-CTF (LRP1-C-terminal fragment). Both extra- and intracellular chains can act independently of each other when the α chain is shed as a soluble LRP1 and the β chain translocates to the nucleus and activates gene transcription and signaling cascades. LPL, lipoprotein lipase; ApoE, apolipoprotein E; COOH, carboxy terminal; EGF, epidermal growth factor; NH2, amino terminal. The image of Figure 1 was modified from the reference (75), adding details to the pictures of the ligands of LRP1.

LRP1, also called CD91 or α-2-M receptor, is a type I glycosylated transmembrane protein comprising a large extracellular domain (ECD) (515 kDa, α chain) and a small intracellular domain (ICD; 85 kDa, β chain), which are non-covalently associated on the cell surface. The large ECD contains four clusters of complement-like repeats and epidermal growth factor (EGF) repeats. The small ICD contains one YXxL motif, two dileucine motifs responsible for endocytosis and two NPxY motifs that function as secondary endocytosis signals and binding sites for signaling adaptor proteins (10–12, 18, 62, 64–69). The NPxY motif can be phosphorylated on several serine, threonine or tyrosine residues to regulate signal transduction (70–72). Additionally, LRP1 can undergo intramembrane proteolysis called regulated intramembrane proteolysis (RIP), which induces cleavage of the released ICD of LRP (LRP-ICD) by γ-secretase and subsequent translocation of this domain into the cell nucleus, where it represses interferon-γ promoter activity to suppress inflammation (73, 74). Like all members of the LDL receptor family, LRP1 consists of five modular structural units: cysteine-rich complement-type repeats (CRs), EGF precursor repeats, β-propeller (YWTD) domains, a transmembrane domain and a cytoplasmic domain (Figure 1) (75). The EGF precursor consists of two cysteine-rich EGF repeats, a YWTD repeat and another EGF-like repeat (76). The extracellular region contains four ligand-binding domains (clusters I-IV) consisting of 2, 8, 10, and 11 CRs, respectively, with clusters II and IV are the major ligand-binding regions (77).

#### sLRP1: The Soluble Form of LRP1

Similar to a wide variety of receptors and other plasma membrane proteins, LRP1 can be cleaved by cell surface proteases such as hepatic metalloproteinases, tPA and neuronal β-secretase protease (BACE1) to produce soluble LRP1 (sLRP1), which can be detected in plasma and cerebrospinal fluid (19, 78–84). This process can be accelerated by inflammatory mediators in cultured bone marrow macrophages, such as lipopolysaccharide (LPS) and interferon-γ (IFN-γ); sLRP1 contains the α chain and a 55-kDa fragment of the β chain, suggesting that cleavage occurs near the plasma membrane (85, 86). In addition, sLRP1 maintains the ligand-binding properties of cellular LRP1 and may therefore act as a competitive ligand uptake inhibitor that binds LRP1 on the cell surface (87). By binding extracellular ligands interacting with LRP1, sLRP1 could regulate their endocytosis or control multiple cell signaling pathways. In RAW 264.7 macrophage-like cells, sLRP1 was shown to promote tumor necrosis factor-α (TNF-α), monocyte chemoattractant protein type-1 (MCP-1)/CCL2 and IL-10 expression through activation of the MAPK and JNK cellular pathways to regulate the inflammatory response (85). These results suggest that sLRP1 may modulate regulatory cytokine expression by macrophages to regulate inflammation or monocyte chemotaxis.

Furthermore, sLRP1 has been demonstrated to be associated with inflammation (85, 88, 89), and increased levels of circulating sLRP1 have been observed shown to be increased in patients with rheumatoid arthritis (RA) or systemic lupus erythematosus (SLE) (85). Several studies have also shown that sLRP1 can mediate Aβ clearance from the brain to the bloodstream via transcytosis across the BBB (90–92), leading to the proposal that sLRP1 could also be used as a biomarker for Alzheimer's disease (93). In addition, the circulating sLRP1 concentration has been reported to be significantly higher in patients with severe hypercholesterolemia than in those with moderate hypercholesterolemia and normocholesterolemic controls. Moreover, as a robust and direct association between sLRP1 and lipid parameters, sLRP1 may be useful as a biomarker for atherosclerosis (94). Another study revealed that sLRP1 is a novel biomarker for the P2Y12 receptor expression, which can aggravate atherosclerosis, in atherosclerotic plaques (95). However, additional studies are needed to confirm the function of sLRP1 *in vivo*.

### Distribution of LRP1

LRP1 is ubiquitously expressed by multiple cells, including VSMCs (17, 96, 97), macrophages (17, 96, 97), hepatocytes (30, 67, 97), epithelial cells (18), retinal Müller glial cells (18), neurons (90, 92, 97), astrocytes (97), fibroblasts (96, 97), adipocytes (54, 98), tumor cells (68), endothelial cells (96, 99), neutrophils (100) and T cells (101). In this review, we primarily discuss the role of LRP1 primarily in cells associated with atherosclerosis.

### Ligands Binding to LRP1

Through its ECD, LRP1 can bind with high affinity and internalize more than 75 distinct, structurally and functionally unrelated ligands, such as proteins involved in lipoprotein metabolism and transport (30, 31, 59, 102–105), proteins involved in AD (59, 90, 91, 106), proteases and protease/inhibitor complexes (27, 31, 34, 57, 107–120), growth factors (121–123), extracellular matrix proteins (124–126), infection (127–130), transcription activation (131), chaperones (132–136), blood coagulation (137, 138) and others (103, 139–157) (Table 1). In addition, these ligands may compete with each other for binding, such as the receptor-associated protein (RAP), a 39-kDa molecular chaperone (77), which can tightly bind to and arrest the binding to LRP1 of some ligands, including tissue-type plasminogen activator (t-PA), thrombospondin (TSP), plasminogen activator inhibitor-1 (PAI-1), midkine (MK), β2-integrins and connective tissue growth factor (CTGF) (100, 122, 123, 133, 158, 159). LRP1 can regulate the cell surface abundance of other membrane proteins, some of which have cell signaling activity, by binding and facilitating their delivery to lysosomes for subsequent degradation (87, 160, 161). Thus, based on the broad spectrum of ligands that it recognizes, LRP1 participates in multiple physiological and pathological processes, including lipid and glucose metabolism, protein degradation, wound healing and tissue repair, cell differentiation, hepatic steatosis, kidney fibrosis, acute respiratory distress syndrome (ARDS), Alzheimer's disease, tumor growth and progression, atherosclerosis, and inflammation (10, 64, 68, 159, 162). The LRP1 cytoplasmic domain can also interact with numerous signaling adapter proteins (163–167) (Table 2), including Shc, disabled protein 1 (Dab1) and Fe65, which are involved in directing cellular trafficking and in cell signaling events.

**Table 1:** Ligands that binds to extracellular domain of LRP1.

**Molecule**	**Function**	**References**
**Lipoprotein metabolism and transport**		
Apolipoprotein E-enriched lipoproteins	Fat-binding protein produced by astrocytes, essential for the catabolism of lipoproteins and their transport; main cholesterol carrier in the brain	(59)
Lipoprotein lipase	Lipase involved in lipoprotein metabolism and transport	(31, 102)
Sphingolipid activator protein	Involved in glycosphingolipid catabolism	(103)
Saposin (SAP) precursor	Glycoprotein precursor of saposins (sphingolipid activator proteins) involved in glycosphingolipid catabolism	(103)
Hepatic lipase	Lipase involved in lipoprotein metabolism and transport	(104)
Trigliceride-rich lipoproteins (TLRs)	Main carriers of triglycerides in the blood; involved in lipoprotein metabolism and transport	(105)
Chylomicron remnants	Lipoprotein particles comprising triglycerides, phospholipids, cholesterol, and proteins involved in lipid transport	(30)
**Protein involved in AD**		
Apolipoprotein E-enriched lipoproteins	Fat-binding protein produced by astrocytes, essential for the catabolism of lipoproteins and their transport; main cholesterol carrier in the brain	(59)
Amyloid β peptide	Peptide derived from amyloid precursor protein processing. Main component of amyloid plaques found in Alzheimer's patients	(90, 91)
Amyloid precursor protein (APP)	Integral membrane protein, during its proteolysis the amyloid β peptide is generated	(106)
**Proteases and protease/inhibitor complexes**		
uPA/C inhibitor complexes	Serine protease–protease inhibitor complex	(27)
Thrombin: protein inhibitor C complexes	Serine protease–protease inhibitor complex	(27)
uPA/PAI-1 complexes	Serine protease–protease inhibitor complex	(31)
tPA/PAI-1 complexes	Serine protease–protease inhibitor complex	(31)
Urokinase-type plasminogen activator (uPA)	Serine protease, involved in tissue remodeling, wound healing, cell migration	(31)
Tissue factor pathway inhibitor (TFPI)	Single-chain polypeptide that reversibly inhibits coagulation factor Xa, thereby regulating blood clotting	(31)
High-temperature requirement factor A1 (HtrA1)	Degrade several matrix components including decorin, fibronectin, aggrecan, type II collagen	(34)
Proteinase 3 (P3)	Regulate cell proliferation	(101)
Aprotinin	Single-chain globular polypeptide derived from bovine lungs; inhibits serine proteases	(107)
Thrombin/anti-thrombin III complex	Serine protease–protease inhibitor complex	(108)
Thrombin: heparin cofactor II complexes	Serine protease–protease inhibitor complex	(108)
α1-antitrypsin/trypsin complexes	Serpin-enzyme complex	(108)
Neuroserpin/tPA complexes	Serine protease–protease inhibitor complex	(109)
uPA/Nexin-1 complexes	Serine protease–protease inhibitor complex	(110)
Thrombin/protease nexin-1 complex	Serine protease–protease inhibitor complex	(111)
Thrombin: PAI-1 complexes	Serine protease–protease inhibitor complex	(112)
Tissue inhibitors of matrix metalloproteases (TIMPs)	Protease inhibitors of matrix metalloproteinases	(113)
Matrix metalloproteinase 2 (MMP-2)	Proteinase involved in the degradation of the extracellular matrix, metastasis	(113)
Matrix metalloproteinase 9 (MMP-9)	Proteinase involved in the degradation of the extracellular matrix, angiogenesis, metastasis	(113)
Matrix metalloproteinase 13 (collagenase-3) (MMP-13)	Proteinase involved in the degradation of the extracellular matrix, angiogenesis, metastasis	(113)
Plasminogen activator inhibitor (PAI-1)	Serpin, Regulator of tPA/uPA activity	(31, 113)
α1-antitrypsin (or A1PI)	Member of the serpin superfamily, inhibits various proteases, regulates enzymes produced by inflammatory cells like neutrophil elastase	(114)
Nexin-1	Member of the serine protease inhibitor (Serpin) superfamily	(115)
Pro-urokinase	Serine protease, urokinase-type plasminogen activator single-chain zymogen with little intrinsic enzymatic activity	(116)
Coagulation factor Xa/tissue factor pathway inhibitor (TFPI) complexes	Coagulation factor X is a serine protease that in its active form (Xa) converts prothrombin into thrombin and plays a role in blood coagulation; TFPI reversibly inhibits factor Xa	(117)
Coagulation factor XIa/protein nexin complexes	Coagulation factor XI is a serine protease that in its active form (XIa) initiates the intrinsic pathway of blood coagulation by activating factor IX; complexes with nexin-1 inhibit its function	(118)
Cathepsin D	Lysosomal aspartic protease, member of the peptidase A1 family, involved in protein degradation	(119)
Pregnancy zone protein (PZP):protease complexes	PZP is a member of the a-2 globulin family; protease inhibitor and extracellular chaperone; role in immune regulation during pregnancy	(120 )
**Growth factors**		
Platelet-derived growth factor (PDGF)-BB, PDGF receptor (PDGFR) β	PDGF-BB is a dimeric glycoprotein composed of two B subunits and a major growth factor that binds with high affinity to the cell surface receptor PDGFR β	(121)
Transforming growth factor-b 1 (TGF-β1)	Multifunctional growth factor, involved in interactions with extracellular proteins, cell growth, differentiation and vascular remodeling	(121)
Transforming growth factor-b 2 (TGF-β2)	Multifunctional growth factor, involved in interactions with extracellular proteins, cell growth, differentiation and vascular remodeling	(121)
Connective tissue growth factor (CTGF; CCN2)	Matricellular protein of the extracellular matrix-associated heparin-binding protein family, involved in cell adhesion, migration, and angiogenesis	(122)
Midkine (MDK)	Heparin-binding growth factor induced during mid-gestation involved in cell migration, survival and angiogenesis	(123)
**Matrix protein**		
Thrombospondin 1	Extracellular matrix glycoprotein, member of the thrombospondin family, vital for cell-cell and cell-matrix interactions	(124, 159)
Thrombospondin 2	Extracellular matrix glycoprotein, member of the thrombospondin family, vital for cell-cell and cell-matrix interactions	(125, 159)
Fibronectin	Glycoprotein of the extracellular matrix vital for cell differentiation, migration and adhesion	(125)
**Infection**		
C1s/C1q	Form the complement component C1 complex that initiates the classical pathway of component activation	(127)
C4b-binding protein (C4BP)	Inhibitor in the complement system	(128)
Complement component 3	Plays a role in the activation of the classical and alternative complement activation pathways	(130)
β2-integrins	Leukocyte adhesion to the vascular wall and subsequent migration to inflammatory sites	(100)
Amidoglycosides: gentamicin, polymixcinB	Antibiotics used to treat various bacterial infections	(129)
**Transcriptional activation**		
HIV-Tat protein	Transactivator of viral genes in cells infected with HIV	(131)
**Chaperone**		
Heat shock protein 90, 96, and 70	Intracellular chaperon proteins assisting in protein folding	(132)
Receptor-associated protein (RAP)	Endoplasmic reticulum resident chaperone glycoprotein, inhibits binding of some ligands to low density lipoprotein receptor family members	(133–135)
Calreticulin	Calcium-binding chaperone protein, regulates many cellular processes	(136)
**Blood coagulation**		
Coagulation factor VIII	Blood-clotting protein, participate in blood coagulation	(137)
Von Willebrand factor (vWF)	Adhesive, glycoprotein involved in blood coagulation and wound healing	(138)
**Others**		
Annexin VI	Member of the calcium-dependent membrane and phospholipid binding	(139)
	proteins; co-receptor of Lrp1, involved in endocytosis processes, interacts with a-2-M	(140)
CCN1, cysteine-rich angiogenic inducer 61 (CYR61)	Secreted, matrix-associated signaling protein involved in apoptosis, adhesion, migration and vascular integrity	(141)
Decorin (Dcn)	Member of the small leucine-rich proteoglycan family that impacts the activities of growth factors, regulates extracellular matrix assembly and cell adhesion	(142)
Glypican-3: Hedgehog complexes	Glypican-3 is a heparan sulfate proteoglycan that impacts embryonic growth by inhibiting the hedgehog signaling pathway	(143)
Heparan sulfate proteoglycans (HSPGs)	Glycoproteins containing one or more covalently attached heparan sulfate chains; present at the cell surface and in the extracellular matrix; endocytic and adhesion receptors, regulate cell migration	(144)
Insulin	Peptide hormone produced by the pancreas that regulates the metabolism of carbohydrates, fats and proteins	(145)
Insulin-like growth factor-binding protein 3 (IGFBP-3)	Protein produced and secreted by the liver, carrier of insulin-like growth factors	(146)
Lactoferrin	Multifunctional protein of the transferrin family with an antibacterial function	(147)
Leptin	Hormone produced by adipose cells involved in energy balance and neuronal functioning	(148)
Malaria circumsporozoite protein (CSP)	Secreted protein of the sporozoite stage of the malaria parasite	(149 )
Metallothionein II Minor-group human rhinovirus (HRV2) Myelin-associated glycoprotein (MAG) Myelin basic protein (MBP) Prion protein (PrP) Pseudomonas exotoxin A Ricin A Saporin TpeL	Cysteine-rich low molecular weight metallothionein family member involved in protection against oxidative stress and chemotactic signal transduction Minor group rhinovirus Cell membrane glycoprotein involved in myelination Major protein forming the myelin sheath of oligodendrocytes and Schwann cells Cell-surface glycoprotein that upon conversion can cause prion diseases Toxin from Pseudomonas aeruginosa Ribosome-inactivating protein found in the seeds of Ricinus communis; potent toxin Ribosome-inactivating protein found in the seeds of Saponaria officinalis; potent toxin Clostridium perfringens toxin	(150) (151) (152) (153) (154) (155) (156) (157)

**Table 2:** Adaptor proteins known to bind to the cytoplasmic domain of LRP1.

**Adaptor proteins**	**Function**	**References**
Disabeled-1 (Dab1)	Src activation, neuronal migration	(163)
FE65	Actin remodeling, APP processing	(164)
Shc	Signal transduction by protein-tyrosine kinases	(164)
PKCα	Proliferation, apoptosis, differentiation, and motility	(165)
Talin-like protein	Coupling to actin cytoskeleton	(165)
OMP25	Mitochondrial transport	(165)
ICAP1	Integrin-mediated signaling	(165)
PSD95	synapse stability, Coupling to NMDA receptors	(165)
SEMCAP-1	Axon guidance, vesicular transport	(165)
JIP1, JIP2	Regulation of MAPK and SAPK, including JNK	(165)
GULP	Phagocytosis	(166)
Cbl	E3 ligase, receptor tyrosine kinase downregulation	(167)

## The Dual Roles of LRP1 in Animal Models of Atherosclerosis

Numerous genetic studies have demonstrated the dual roles of LRP1 in atherosclerosis-related cells in different animal models (Table 3). LRP1 gene deletion promotes the progression of atherosclerosis for progressive plaques, while promotes the regression of atherosclerosis for established plaques. The generation and analysis of animal models with receptor gene deletion have elucidated important functions of LRP1 in lipoprotein metabolism and significantly advanced our understanding of the pathophysiological process in patients with lipid disorder.

**Table 3:** Animal experiments about effects of deletion of LRP1 on atherosclerosis lesions.

**Model^a^**	**Control**	**Number**	**Results (Model vs. Control)**	**References**
LDLR^−/−^/smLRP^−/−^+HFD	smLRP^+/+^/LDLR^−/−^	NA	Disruption of the elastic layer, SMC proliferation, aneurysm formation, and marked susceptibility to cholesterol-induced atherosclerosis, no effect on plasma cholesterol or triglyceride levels	(14)
smLRP^−/−^	smLRP^+/+^ littermates	46	Greater smooth muscle cell proliferation, deficient contractile protein expression, impairment of vascular contractility, and promotion of denudation-induced neointimal hyperplasia	(33)
LDLR^−/−^/smLRP^−/−^+HFD	smLRP^+/+^/LDLR^−/−^ littermates	44	Disruption of elastic layers, vascular fibrosis, elongation and distension of the aorta, susceptibility to atherosclerosis	(121)
smLRP^−/−^+CD	smLRP^+/+^ littermates	NA	Increased total cholesterol levels and reduced ABCA1 protein expression in the aorta, increased cellular lipid accumulation is detected in LRP1-deficient SMCs	(187)
macLRP1^−/−^LDLR^−/−^+WD	macLRP1^+/+^/LDLR^−/−^	NA	40% increase in atherosclerosis in proximal aorta, increase monocyte chemoattractant protein type-1, tumor necrosis factor-α, and proximal aorta macrophage cellularity and matrix metalloproteinase-9	(36)
macLRP^−/−^/apoE^−/−^/LDLR^−/−^+CD	macLRP1^+/+^/apoE^−/−^/LDLR^−/−^ littermates	48	1.8-fold increase in total atherosclerotic lesion area that contained more collagen and less CD3+ T cells	(37)
apoE^−/−^/macLRP1^−/−^+WD LDLR^−/−^ apoE^−/−^/macLRP1^−/−^+WD	apoE^−/−^macLRP1^+/+^ LDLR^−/−^ apoE^−/−^/macLRP1^+/+^	107	163% more Oil-Red-O and 133% more MOMA-2 staining in the proximal aorta, lesion necrosis increased by 6 fold, decreased efferocytosis and 3.5-fold increase in apoptotic cells in lesions, the lesions contained 3.6-fold more Ly6-C positive cells and 2.2-fold more CCR2-positive cells; 88% more lipid-stainable lesion in the proximal aorta and 138% increase in MOMA-2 stainable intimal macrophages.	(39)
macLRP^−/−^ LDLR^−/−^/macLRP^−/−^	macLRP1^+/+^ LDLR^−/−^/macLRP^+/+^	16	Increase IL-1, IL-6, and tumor necrosis factor expression, impair efferocytosis and promotes necrosis	(63)
macLRP1^−/−^LDLR^−/−^+WD+ adalimumab (TNFα inhibitor)	macLRP1^+/+^/LDLR^−/−^+adalimumab	5–7/group	Negates the anti-atherosclerotic benefits of anti-TNFα inhibitor adalimumab	(168)
ApoE^−/−^macLRP1^−/−^+HFD	ApoE^−/−^macLRP1^+/+^	9–11/group	Accelerates atherosclerosis regression, enhances RCT, and increases expression of the motility receptor CCR7 to drive macrophage egress from lesions	(169)
LRP1^f/f^/Tie2Cre+	LRP1^f/f^/Tie2Cre-littermates	18	Increase angiogenesis, endothelial cell proliferation and cell cycle progression	(205)
LRP1^f/f^/Tie2Cre+ +CC or HFD	LRP1^f/f^/Tie2Cre-littermates	44	Regulate global energy homeostasis and alleviate obesity and insulin resistance	(209)
adLRP1^−/−^+WD	adLRP1^+/+^	NA	3-fold increase in atherosclerosis and the adipocytes were smaller, adipose tissues were more inflamed with increased monocyte–macrophage infiltration and inflammatory gene expression	(98)
adLRP1^−/−^+HFD	adLRP1^+/+^ littermates	49	Delayed postprandial lipid clearance, reduced body weight, smaller fat stores, lipid-depleted brown adipocytes, improved glucose tolerance and elevated energy expenditure	(213)

*smLRP^−/−^, smooth muscle–specific LRP1 inactivation; macLRP^−/−^, macrophage–specific LRP1 inactivation; adLRP1^−/−^, adipocyte-specific LRP1 knockout; LRP1 ^f/f^//Tie2Cre+, endothelial cell-specific LRP1-deficient; HFD, high-fat diet; WD, western-type diet; CC, control chow; CD, chow diet; RCT, reverse cholesterol transport; CCR7, CC-chemokine receptor 7; NA for not available*.

a *The animals used for model are mice*.

### LRP1 Gene Deletion Facilitates the Development of Atherosclerotic Lesions

The effect of LRP1 on the progression of atherosclerosis has been tested in ApoE–/– and LDLR–/– mice, with the results showing that atherosclerosis development was enhanced in ApoE–/–, LDLR–/– and ApoE/LDLR double knockout mice harboring LRP gene deletions. Hu et al. reported that macrophage-specific LRP-deficient mice in an ApoE/LDLR double-deficient background showed a 1.8-fold increase in total atherosclerotic lesion area in the aortic root that was accompanied by a 1.7-fold increase in collagen content and a 2.3-fold decrease in the number of CD3+ T cells in lesions (37). Similarly, another study showed that macrophage LRP deletion in the LDLR-deficient mouse model enhanced atherosclerosis development and increased monocyte chemoattractant protein type-1 (MCP-1), TNFα, and matrix metalloproteinase-9 (MMP-9) levels as well as proximal aorta macrophage cellularity (36). Another study showed a similar result that specific deletion of macrophage LRP1 in the ApoE deficient mice increased atherosclerosis, which is concomitant with the accumulation of apoptotic cells and proinflammatory monocytes in lesions (39). Moreover, the inactivation of LRP1 in VSMCs in LDLR–/– mice resulted in disruption of the elastic layer and marked susceptibility to atherosclerosis together with platelet-derived growth factor (PDGF) signaling pathway overactivation (14, 121). Furthermore, LRP1 deficiency in macrophages led to an increase in cell death and inflammation and abolished the antiatherosclerotic benefits of the antitumor necrosis factor-α (TNFα) inhibitor adalimumab (63, 168). A similar result was observed in adipocytes, where adipocyte-specific LRP1 knockout (adLRP1–/–) mice fed a western diet for 16 weeks exhibited a 3-fold increase in atherosclerosis and enhanced inflammation in adipose tissues compared to adLRP1+/+ mice (98).

### LRP1 Gene Deletion Accelerates Atherosclerosis Regression in Mice

In 2018, Paul et al. reported that macrophages (MΦLRP1–/–) can promote atherosclerosis regression independent of plasma lipid levels, increase reverse cholesterol transport (RCT) and cause selective loss of inflammatory M1 macrophages. In this study, ApoE–/– mice were fed a high-fat diet for 12 weeks, and then reconstituted with bone marrow from apoE-producing wildtype (WT) or MΦLRP1–/– mice, then fed a chow diet for 10 weeks. The results showed that MΦLRP1–/– recipients showed 13% smaller plaques, 1.4-fold higher reverse cholesterol transport (RCT), 36% fewer M1 macrophages and 2.5-fold more CCR7+ macrophages in the plaques than those of WT recipients (169).

## Molecular Mechanism of LRP1 in Atherosclerosis-Associated Cells

### LRP1 in VSMCs

**Figure 2:**
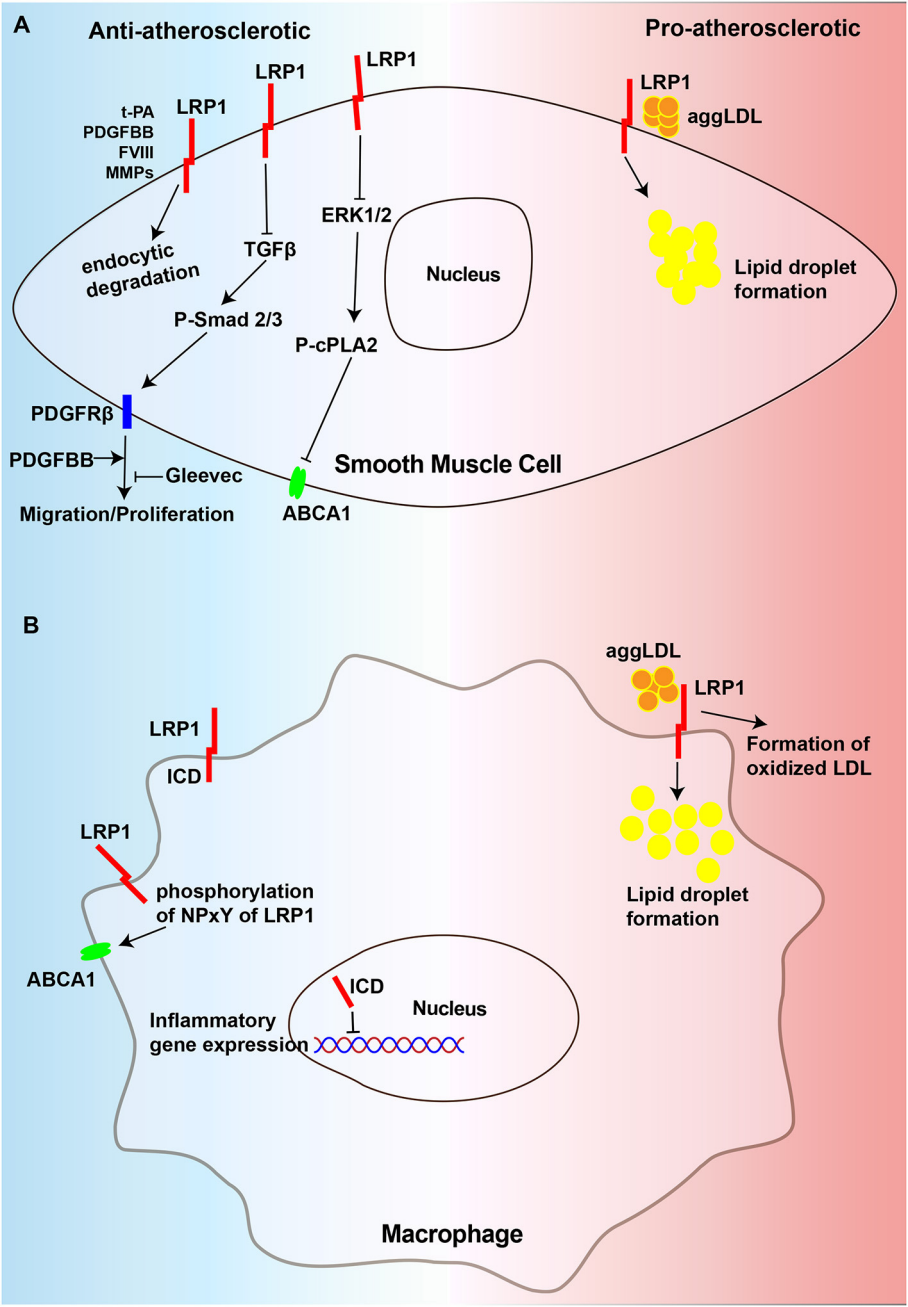
Diagram of the mechanism of regulating atherosclerosis of LRP1 in VSMCs and macrophages. **(A)** LRP1 in VSMCs could degrade extracellular PDFGBB, MMPs, t-PA, and FVIII, which are atherogenic factors, in the way of endocytosis to play the role of anti-atherosclerosis. It could inhibit migration and proliferation of VSMCs to resist atherosclerosis by preventing the activation of TGFβ/Smad2/3/PDGFRβ signaling pathway. Gleevec could also has the effect to inhibit PDGFRβ. Additionally, LRP1 inhibit the activation of ERK1/2 which could suppresses the expression of ABCA1 by promoting phosphorylation of cPLA2. In contrast, LRP1 in VSMCs accelerates the development of atherosclerosis by promoting the uptake of aggLDL and the formation of foam cells. **(B)** The LRP1 can also be cleaved by γ-secretase to release a 25-kDa β-chain fragment, which translocates to the nucleus to suppress inflammatory gene expression. The phosphorylation of NPxY of LRP1 ICD promotes the expression of ABCA1. Nevertheless, LRP1 in macrophages accelerates the development of atherosclerosis by promoting the uptake of aggLDL and the formation of oxidized LDL. AS, atherosclerosis, ABCA1, ATP-binding cassette transporter A1, cPLA2, cytosolic phospholipase A2.

VSMCs are the primary cell type in the vessel wall and a major component of atherosclerotic plaques at all stages (170, 171). VSMCs can also generate extracellular matrix to form the fibrous cap and hence stabilize plaques, yet aberrant VSMCs proliferation promotes atherosclerotic plaque formation (9). The results of some studies indicated that in VSMCs, LRP1 helps to suppress atherosclerosis by inhibiting the platelet-derived growth factor (PDGF) signaling pathway (14, 121). However, the opposite result has been observed in other studies, indicating that LRP1 mediates aggLDL uptake to induce high intracellular cholesteryl ester accumulation in VSMCs, causing the formation of VSMC-derived foam cells and promoting atherosclerosis progression (44–46). Thus, in VSMCs, LRP1 may exert two different and opposing effects on atherosclerosis (Figure 2A).

#### LRP1 in VSMCs Protects Against Atherosclerosis

VSMCs are induced to proliferate and migrate from the media to the intima, contributing to the development of atherosclerosis and restenosis under pathological conditions (9, 32, 172). The binding of PDGF, a potent mitogen for fibroblasts and VSMCs, to PDGF receptor β (PDGFRβ) activates a signaling pathway that promotes VSMs growth and migration, which are crucial in atherosclerosis and neointima formation and LRP1 has been proven to suppress this process through incompletely understood mechanisms (14, 24, 33, 121, 173–175). Studies have elegantly demonstrated this effect via the generation of smLRP1–/– mice on a background of LDLR deficiency, showing that smLRP1–/–/LDLR–/– mice are not only more susceptible to cholesterol-induced atherosclerosis than LDLR–/– mice but also exhibit PDGFR overexpression and increased phosphorylation of Smad2, a downstream component of the TGFβ pathway (14, 121). Moreover, the PDGF-BB pathway has previously been described as a target of the TGFβ signaling pathway (176–178). In addition, LRP1 is identical to TGFβ receptor (V), which is a member of the TGFβ receptor superfamily and is expressed on the cell surface together with TGFβ receptors I, II, and III (179). Therefore, the TGFβ activation pathway in the absence of LRP1 could further activate the PDGF-BB signaling pathway by increasing PDGFRβ expression in the arterial wall, promoting the formation of atherosclerotic lesions. Indeed, the vascular pathology observed in smLRP1–/–/LDLR–/– mice was significantly improved by blockade of the PDGF or TGFβ receptor signaling cascades with the tyrosine kinase inhibitor Gleevec (121). Overall, the results of these studies reveal that in VSMCs, LRP1 plays a major role in maintaining the integrity of the vascular wall and reducing atherosclerosis by suppressing the PDGFRβ and TGFβ signaling pathways.

The role of the intracellular NPxYxxL motif of the LRP1-ICD in the development of atherosclerosis has attracted increasing attention. One study has thoroughly demonstrated that inactivation of the LRP1 NPxYxxL motif in LDLR–/– mice showed a significant 1.5-fold increase in the development of atherosclerosis compared to that observed in LDLR–/– control mice. MMP2 activity, which could be degraded by lysosomal proteases after being endocytically taken by LRP1 (113, 180), as well as could facilitate VSMC migration and plaque rupture by degrading the extracellular matrix (181, 182), showed a significant 2.7-fold increase in the aortas of NPxYxxL-inactivated mice. The results of this study also indicated that there was a significant 2-fold increase in the number of apoptotic cells relative to the plaque size in mice with the NPxYxxL-inactivation mice, which was caused by the secretion of the proapoptotic cytokine TNF-α. Therefore, we can conclude that the intracellular NPxYxxL motif of the LRP1-ICD is essential for the atheroprotective role of LRP1 (183). LRP1 can also protect against atherosclerosis by regulating the expression of ATP-binding cassette transporter A1 (ABCA1). The results of multiple studies have indicated that increased mitogenic signaling in the absence of LRP1 can regulate ERK1/2 activation, leading to increased cytosolic phospholipase A2 (cPLA2) phosphorylation, which in turn promotes the production of arachidonic acid (14, 184, 185), a suppressor of LXR-driven ABCA1 expression (186). This cascade reduces cholesterol efflux from VSMCs and promotes the formation of foam cells (187). In summary, LRP1 plays an antiatherosclerotic role in VSMCs, and its absence promotes atherosclerosis development (Figure 2A).

#### LRP1 in VSMCs Promotes the Development of Atherosclerosis

In addition to protecting against atherosclerosis, many studies have also shown that LRP1 overexpression is associated with atherosclerosis progression in both animal (188) and human models of atherosclerosis (17, 97, 189). LRP1 can promote the formation of VSMC-derived foam cells, leading to the progression of atherosclerosis. In addition, its expression is upregulated by lipid accumulation during the progression of atherosclerotic lesions in humans, and VSMCs derived from advanced atherosclerotic plaques show higher intracellular lipid deposition than those from less-advanced plaques due to their higher LRP1 expression levels (190). In addition, the results of another study also indicated that the transient receptor potential vanilloid type-1 (TRPV1) activation-induced decrease in LRP1 expression reduces lipid uptake by VSMCs (191). Several studies have shown that LRP1 mediates the uptake of aggLDL and induces adipose differentiation-related protein (ADRP) overexpression, leading to an increase in intracellular lipid deposition in VSMCs. ADRP is localized on the surface monolayer of lipid droplets and is considered a specific marker of lipid droplet formation (41, 42, 44–46, 189, 192, 193). Moreover, aggLDL can further upregulate LRP1 expression in VSMCs in a time- and dose-dependent manner (48). This positive feedback mechanism is highly efficient in promoting the formation of VSMCs foam cells. The formation of VSMC-derived foam cells, a main proatherogenic mechanism of LRP1, is associated with atherosclerotic lesion progression. Therefore, in VSMCs, LRP1 can also promote atherosclerosis development by promoting the formation of foam cells (Figure 2A).

### LRP1 in Macrophages

Macrophages are the most abundant type of immune cells in atherosclerotic lesions, playing an important role in all stages of atherosclerosis, from the formation of atherosclerotic lesions to plaque rupture (194). Lipoprotein receptors in macrophages can accelerate the progression of atherosclerosis by facilitating the uptake of atherogenic particles, such as oxidized lipoproteins, and inducing vascular inflammation (172, 195, 196). However, the role of macrophage LRP1 in atherosclerosis is controversial, as macrophage LRP1 not only protects against but also promotes atherosclerosis (Figure 2B).

#### LRP1 in Macrophages Protects Against the Development of Atherosclerosis

Macrophage LRP1 has been shown to exert atheroprotective effects. However, mice macrophage-specific LRP deficiency in an apoE/LDLR double-deficient background did not exhibit altered plasma lipid levels or plasma lipoprotein profiles but did exhibit an increase in the total atherosclerotic lesion area (37). Additionally, Overton et al. showed that LDLR–/– mice transplanted with bone marrow from macLRP1–/– mice were 40% greater in proximal aorta lesions than that observed in high-fat diet-fed mice transplanted with control bone marrow, and accompanied by the increase in proinflammatory factors, such as monocyte chemoattractant protein type-1 (MCP-1) and TNF-α, but en face analysis of the distal aorta showed no significant difference likely attributable to the shor-term experiments (36). Furthermore, atherosclerotic lesions in mice lacking LRP1 expression in macrophages are characterized by increased apoptosis, suggesting that LRP1 prevents atherosclerosis by promoting efferocytosis to remove apoptotic cells from plaques, which is also manifested as inhibition of the p-AKT survival pathway and the promotion of inflammation with increased IL-1, IL-6 and TNF-α expression (63). Furthermore, LDLR–/– mice that received lethal irradiation and were reconstituted with bone marrow from MΦLRP1–/– mice fed on a western-type diet for 10 weeks showed increased necrosis and apoptosis, defective efferocytosis and increased inflammation in the lesions (168). Another study showed that the specific deletion of macrophage LRP1 in ApoE–/– mice promoted atherogenesis and apoptotic cell accumulation in lesions, partially due to decreased efferocytosis and increased lesion necrosis compared to ApoE–/– mice. In this research, the authors also indicated that 88% more lipid-stainable lesion areas in the proximal aorta and a 138% increase in MOMA-2 stainable intimal macrophages in LDLR–/– mice receiving ApoE–/– macLRP1–/– bone marrow compared with those that receiving ApoE–/– marrow. Aorta en face lesions were not significantly different between ApoE–/– macLRP1–/– and ApoE–/– BM-recipient mice, which is consistent with the previous report in 2007 (39). In addition, LRP-1 can also affect macrophage polarization and promote polarization toward the anti-inflammatory M2 functional phenotype (197), leading to an increase in the number of anti-inflammatory M2 macrophages in lesions. In another study, LRP1 was proposed to inhibit cellular inflammatory responses in an adipocyte-specific LRP1-deficient mouse model (98). Another potential mechanism by which LRP1 affects inflammation in macrophages may be through the direct regulation of inflammatory gene transcription. In cultured macrophages, the LRP1-ICD can be cleaved from the plasma membrane by γ-secretase upon induction by inflammatory mediators, such as LPS and interferon-γ, and then translocate into the nucleus, where it promotes the nuclear export and proteasomal degradation of interferon regulatory factor 3, thereby limiting the expression of the proinflammatory genes in cultured fibroblasts and macrophages (73, 85). Apart from regulating inflammation, a study using a knock-in mouse model of LRP1Y63F, in which the tyrosine in the distal NPxY motif was replaced with phenylalanine to prevent NPxY phosphorylation of LRP1, revealed that LRP1 not only regulates the expression of ABCA1, the major cholesterol exporter, to maintain cholesterol efflux but also integrates cellular cholesterol homeostasis with inflammation and efferocytosis (15). In summary, macrophage LRP1 may primarily exert an atheroprotective effect mainly by decreasing inflammation, facilitating efferocytosis and promoting cholesterol efflux (Figure 2B).

#### LRP1 in Macrophages Promotes the Development of Atherosclerosis

Evidence from several *in vitro* studies shows that LRP1 has proatherogenic properties in macrophages. First, LRP1 is upregulated during macrophage foam cell formation (198). Second, macrophage LRP1 has been shown to play a vital role in the translocation of 12/15-lipoxygenase, promoting the formation of oxidized LDL (199, 200). Third, LRP1 was shown to mediate the internalization of aggLDL and apoE-rich atherogenic lipoproteins, along with LDLR, into macrophages (46, 201, 202). Through the above mechanisms, the accumulation of lipids and macrophage-derived foam cells increases, leading to the progression of atherosclerosis. In addition, the results from an animal study proved that mice with macrophages lacking LRP1 (MΦLRP1–/– mice) exhibit accelerated regression of atherosclerosis and enhanced reverse cholesterol transport (RCT), and drive macrophage egress from lesions by inducing expression of the motility receptor CCR7 (169). Therefore, macrophage LRP1 can also lead to atherosclerosis progression by promoting foam cell formation (Figure 2B).

### LRP1 in Endothelial Cells

Atherosclerosis is a chronic process initiated by endothelial dysfunction and structural changes (203). Although LRP1 is highly expressed in a variety of cells, its protein expression levels in endothelial cells are low (96, 204). However, LRP1 expression is tightly regulated by various physiological conditions in endothelial cells, reflecting its crucial role in these cells. An elegant study showed that LRP1 regulates hypoxia-mediated angiogenesis by inhibiting PARP-1 activity and suppresses endothelial cell proliferation by preventing cell cycle progression in an oxygen-induced retinopathy (OIR) mouse model (205). Another study also showed that LRP1 can mediate bone morphogenetic protein-binding endothelial regulator (BMPER)-mediated bone morphogenetic protein 4 (BMP4) signaling to regulate endothelial cell migration and angiogenesis (206). Angiogenesis is the process of growing new blood vessels from the existing vascular network that occurs during embryonic development and throughout adulthood, and begins under wound healing and pathological conditions, such as retinopathy. Pathological retinal angiogenesis produces physiologically defective blood vessels, leading to exudation and hemorrhage that threatens vision (207). Moreover, a study showed that LRP1-dependent BMPER signaling is required for LPS-induced nuclear factor of activated T cells 1 (NFATc1) activation to induce acute inflammatory responses in endothelial cells (208), which may cause the initiation of atherosclerosis. Interestingly, LRP1 can also promote peroxisome proliferator-activated receptor-γ (PPARγ) activity by acting as a coactivator to regulate lipid and glucose metabolism in endothelial cells (209). Therefore, it can be used to treat pathological retinal angiogenesis induced by diabetic retinopathy and atherosclerosis by regulating the expression and function of LRP1 in endothelial cells.

### LRP1 in Adipocytes

Another cell type with high LRP1 expression and associated with the development of atherosclerosis is adipocytes. An increasing number of studies have shown that adipose tissues, especially those in the perivascular area surrounding the vessel wall, such as the aorta, coronary artery, and carotid artery, also play a vital role in the pathogenesis of atherosclerosis (210, 211). Perivascular adipose tissue (PVAT) is a unique conglomerate of various cell types, including adipocytes, preadipocytes, and mesenchymal stem cells that are embedded in a matrix that is invested with microvessels, and that is important for the maintenance of the vascular structure and the regulation of vascular function and homeostasis (210). The interaction between perivascular adipocytes and vascular wall cells, such as endothelial cells and VSMCs, is essential for normal vascular function, and may be disturbed in diseases such as atherosclerosis. In the early process of hyperlipidemia, atherosclerosis prone animal models and human arteries, PVAT expansion and the production of chemokines near the adventitia of large arteries have been detected, leading to the aggravation of inflammation, which may play a fundamental role in the pathogenesis of the cardiovascular disease (CVD) (212). Adipose tissues have been shown to exhibit increased inflammation with enhanced monocyte–macrophage infiltration and inflammatory gene expression in mice with adipocyte-specific LRP1 knockout (adLRP1–/–) in PVAT, and that mice transplanted with PVAT from adLRP1–/– mice displayed a 3-fold increase in atherosclerosis compared to those transplanted with PVAT from adLRP1+/+ mice after a western diet (98). In addition, mice with LRP1 knocked out in adipocytes (adLRP1–/– mice) exhibited delayed postprandial lipid clearance, reduced fat stores, improved glucose tolerance and decreased body weight compared to wild-type mice (213). Taken together, the results of these studies have added the adipose tissue to the list of anatomic sites where LRP1 expression is important for atheroprotection. Thus, it is possible to prevent the development of atherosclerosis and relieve associated clinical symptoms by regulating LRP1 in adipocytes, including through the use of natural and synthetic LRP1 agonists.

### LRP1 in Immune Cells

#### LRP1 in Neutrophils

LRP1 is also abundantly present in neutrophils, which are the most abundant type of white blood cell in the human circulation and the principal cell type during acute inflammatory reactions (97, 214). The number of circulating neutrophils is a predictor of future adverse cardiovascular events and positively correlates with the size of developing lesions in humans and mice, respectively (215, 216). The expression of endothelial cell adhesion molecules (e.g., E-selectin, P-selectin, and intercellular adhesion molecule-1) increases once the endothelial cell dysfunction is triggered by exposure to irritative stimuli such as hyperlipidemia and proinflammatory cytokines, which then triggers the recruitment of neutrophils. In addition, neutrophils can release chemotactic proteins and proinflammatory mediators to promote monocyte recruitment as well as vascular inflammation, promoting atherosclerosis development. Furthermore, neutrophils can degranulate large amounts of different proteases including matrix metalloproteases (MMPs), myeloperoxidase (MPO) and neutrophil elastase or form neutrophil extracellular traps, leading to a thinner fibrous cap and subsquent plaque rupture (214, 217). The results of one study indicated LRP1 blockade could prevent intravascular adherence and neutrophil recruitment within the ischemic tissue induced by PAI-1 derived from both leukocytes and non-leukocytic sources (158). Another similar study showed that LRP1 synthesized and expressed by neutrophils accounts for the r-tPA-induced migration and degranulation of neutrophils (217), which can aggravate tissue damage and inflammation. Additionally, in a hind limb ischemia model, LRP-1 was observed to act as the receptor of cytokine midkine (MK) to support neutrophil adhesion and trafficking by promoting the high-affinity conformation of β2 integrin, suggesting a role of LRP-1 in acute inflammation (218). Furthermore, another study also demonstrated that LRP1 can bind to β2 integrin complex expressed in neutrophils to regulate the firm adhesion and subsequent transmigration of neutrophils (100). Taken together, the result of these studies indicate that LRP1 in neutrophils primarily plays a role in neutrophil-associated inflammation.

#### LRP1 in T Cells

T lymphocytes account for another majority of immune cells in human atherosclerotic plaques obtained from endarterectomy (219). A large body of evidence from animal studies suggests that the T-cell response is proatherogenic. Apoe–/– mice crossed with mice lacking the V(D)J recombination-activating protein 1 Rag1 (Rag1–/–) or mice with a severe combined immunodeficiency (SCID) mutation (scid/scid mice) are immunodeficient owing to impaired T-cell and B-cell development and showed reduced atherosclerosis lesions when fed a chow diet (220). Poor LRP1 expression in T cells led to suppression of T cell adhesion to fibronectin and ICAM-1 as well as TCR-induced activation, subsequently suppressing accumulation at sites of inflammation (221). Nevertheless, another study showed that LRP1 inhibites the adhesion of T cells to ICAM-1 and fibronectin via JAK signaling (222). Furthermore, LRP1 on T cells interactes with membrane-associated proteinase 3 (mP3) on neutrophils resulting in inhibition of the inflammatory response (101). In summary, LRP1 on T cells may also play a major role in the inflammatory response associated with T cells.

## Conclusions

LRP1 is a large, multifunctional type 1 transmembrane receptor and essential for maintaining basic cellular functions and the development of organisms. Due to its ability to mediate actions of a broad range of ligands, LRP1 participates in the development of multiple degenerative diseases, such as atherosclerosis and Alzheimer's disease. In this review, we summarized the dual and opposing roles of LRP1 in atherosclerosis *in vivo* and *in vitro*. The role of LRP1 in VSMCs and macrophages in the development of atherosclerosis is different and opposite *in vivo* and *in vitro*, which may be the reason that the complexity of cross-talks among various signal pathways and different cell types and organ systems *in vivo*. Probably more surprising, for established plaque, the lack of LRP1 expression in macrophages unexpectedly promotes atherosclerosis regression. As a result, the opposite effect of LRP1 involved in the regulation of atherosclerosis might depend on whether the plaque is growing or shrinking. The dual role of LRP1 in mediating the effect of TNF-α on vascular inflammation and in balancing the effects of CD47 on efferocytosis could be involved in this phenomenon (223, 224). However, in endothelial cells and adipocytes, LRP1 may promote resistance to atherosclerosis, primarily by inhibiting inflammation. These results suggest that the functionality of LRP1 is dependent on the cell type in which this receptor is expressed.

### Therapeutic Considerations

This review may provide a basis for the development of new therapeutic approaches for atherosclerosis that target LRP1 and its downstream cellular signaling pathways. For example, the C-terminal half of the cluster II CR9 domain (Gly1127–Cys1140) has been reported to be crucial for LRP1-mediated aggLDL binding and internalization in human VSMCs (hVSMCs) (45). Moreover, anti-P3 (Gly1127–Cys1140) antibodies (Abs) that specifically block the LRP1 (CR9) domain have been shown to efficiently prevent LRP1-mediated aggLDL internalization and aggLDL-induced LRP1 upregulation to prevent human macrophages and VSMCs from forming foam cell formation (45, 225). These findings indicate that this strategy could be used to prevent the occurrence and progression of atherosclerosis. In terms of established plaques, inhibiting LRP1 in macrophages with blocking antibodies could accelerate plaque regression, potentially alleviating atherosclerosis and related cardiovascular and cerebrovascular complications. Notably, LRP1 can regulate the endocytic clearance of several MMPs (113, 226), which could degrade the extracellular matrix to promote VSMC migration and thin the fibrous cap, causing plaque rupture and leading to myocardial infarction and stroke (227). Therefore, whether the inhibition of LRP1 would disrupt the signaling pathways involved in the proliferation and migration of VSMCs and proteolysis activity of MMPs will require a more comprehensive understanding.

However, some studies have shown that the natural LRP1 agonists SERPINs, such as α-2-macroglobulin (A2MG), α-1-antitrypsin (AAT), antithrombin III, and synthetic LRP1 agonists (SP16) can be used to treat myocardial ischemia-reperfusion injury by inducing cytoprotective signals in cardiomyocytes, such as the activation of Akt- and ERK1/2-dependent prosurvival as well as anti-inflammatory signaling pathways. Moreover, AAT and SP16 are under clinical development, and also there are no treatment-related serious adverse events or toxicity (228, 229). Thus, the natural and synthetic agonists of LRP1 may be useful in treating atherosclerosis by inhibiting inflammation and promoting vascular cell survival, but additional evidence is needed to elucidate the associated mechanism. Gene therapy could be used to increase LRP1 expression. For instance, it may be possible to use adeno-associated virus-2 (AAV-2) carrying the cDNA of LRP1 or its smaller fragments to increase LRP1 expression in vascular cells to resist atherosclerosis. In a mouse model of Alzheimer's disease, treatment with recombinant ligand-binding domain IV of LRP1 by using an *in situ* arterial brain perfusion technique for 3 months reduced brain Aβ levels (90). Therefore, gene therapy and recombinant LRP1 could be used to treat and prevent atherosclerosis.

In summary, in this review, LRP1 was shown to participate in a large number of physiological activities as a coreceptor and to interact with many adaptor proteins through its cytoplasmic domain. The understanding of the mechanisms and the further identification of LRP1 partners may open up new ways to treat metabolic diseases, such as lipid metabolism, atherosclerosis, inflammation, Alzheimer's disease and obesity. Futher investigations will most likely uncover even more functions of these receptor beyond those considered here.

## Author Contributions

LM and BH designed the review. JC, YS, and SP wrote the manuscript. All authors contributed to the article and approved the submitted version.

## Conflict of Interest

The authors declare that the research was conducted in the absence of any commercial or financial relationships that could be construed as a potential conflict of interest.

## Abbreviations

VSMCs: vascular smooth muscle cells
α-2-M: α2-macroglobulin receptor
LRP1: low-density lipoprotein receptor-related protein 1
α-SMA: α-smooth muscle actin
HFD: high-fat diet
BBB: blood-brain barrier
VLDL: very-low-density lipoprotein receptor
aggLDL: aggregated low-density lipoprotein
CEs: cholesteryl esters
ICD: intracellular domain
ECD: extracellular domain
EGF: epidermal growth factor
CRs: cysteine-rich complement-type repeats
cPLA2: cytosolic phospholipase A2
BMPER: bone morphogenetic protein-binding endothelial regulator
NFATc1: nuclear factor of activated T cells 1
MAPK: mitogen-activated protein kinase
IR: insulin receptor
AKT: serine/threonine protein kinase
ERK: extracellular signal-regulated kinase
JNK: c-jun N-terminal kinase
t-PA: tissue-type plasminogen activator
u-PA: urokinase-type plasminogen activator
MMP-9: matrix metalloproteinase-9
MCP-1: monocyte chemoattractant protein type-1
TNFα: tumor necrosis factor-α
TSP: thrombospondin.
