# Corrigendum: The dual role of low-density lipoprotein receptor–related protein 1 in atherosclerosis

**DOI:** 10.3389/fcvm.2023.1179643

**Published:** 2023-04-21

**Authors:** Jiefang Chen, Ying Su, Shulan Pi, Bo Hu, Ling Mao

**Affiliations:** Department of Neurology, Tongji Medical College, Union Hospital, Huazhong University of Science and Technology, Wuhan, China

**Keywords:** LRP1, adipocytes, atherosclerosis, endothelial cells, immune cells, macrophages, smooth muscle cells

A Corrigendum on The dual role of low-density lipoprotein receptor-related protein 1 in atherosclerosis By Chen J, Su Y, Pi S, Hu B, Mao L. (2021) Front Cardiovasc Med. 8:682389. doi: 10.3389/fcvm.2021.682389

**Missing Citation**

In the published article, (75) was not cited in the article. The citation has now been inserted in **[INTRODUCTION TO LRP1]**, **[***Structure of LRP1***]**, [Paragraph one], and the text should read:

“[Like all members of the LDL receptor family, LRP1 consists of five modular structural units: cysteinerich complement-type repeats (CRs), EGF precursor repeats, β-propeller (YWTD) domains, a transmembrane domain, and a cytoplasmic domain (Figure 1) (75).]”

The authors apologize for this error and state that this does not change the scientific conclusions of the article in any way. The original article has been updated.

**Error in Figure Legend**

In the published article, there was an error in the legend for (Figure 1) as published. (A note regarding the original image of Figure 1 was missing.) The corrected legend appears below.

[FIGURE 1 Structure of mature low-density lipoprotein receptor–related protein-1 (LRP-1). LRP1 is a multifunctional receptor that binds a large spectrum of extracellular and intracellular ligands. The α chain contains long modular arrays of acidic cysteine-rich complement-type repeats (CRs), along with epidermal growth factor (EGF)-like domains and β-propeller modules. Also, it consists of four ligand-binding regions (I, II, III, and VI), which are composed of 2, 8, 10, and 11 CRs, respectively. The cytoplasmic tail contains two NPxY motifs that are required for endocytosis and multiple signaling pathways. LRP1 has an YXXL motif adjacent to the second NPxY motif, enabling rapid endocytosis. The β chain also interacts with scaffolding proteins such as PSD-95, Dab-1, and FE-65. Regions II and IV bind most of the currently mapped known ligands of LRP1. β-Secretase (BACE1) cleaves the extracellular domain of LRP1 to form sLRP1 and LRP1-CTF (LRP1-C-terminal fragment). Both extra- and intracellular chains can act independently of each other when the α chain is shed as a soluble LRP1 and the β chain translocates to the nucleus and activates gene transcription and signaling cascades. LPL, lipoprotein lipase; ApoE, apolipoprotein E; COOH, carboxy terminal; EGF, epidermal growth factor; NH2, amino terminal. The image of Figure 1 was modified from the reference (75), adding details to the pictures of the ligands of LRP1.]

The authors apologize for this error and state that this does not change the scientific conclusions of the article in any way. The original article has been updated.

## References

[75] Emonard H Théret L Bennasroune AH Dedieu S. Regulation of LRP-1 expression: make the point. Pathol Biol (Paris). (2014) 62(2):84–90. 10.1016/j.patbio.2014.02.002 24661974

